# Remapping high-capacity, pre-attentive, fragile sensory memory

**DOI:** 10.1038/s41598-017-16156-0

**Published:** 2017-11-21

**Authors:** Paul Zerr, Surya Gayet, Kees Mulder, Yaïr Pinto, Ilja Sligte, Stefan Van der Stigchel

**Affiliations:** 1 Experimental Psychology, Helmholtz Institute, Utrecht University, Utrecht, The Netherlands; 2 Methodology and Statistics, Utrecht University, Utrecht, The Netherlands; 3 Brain and Cognition, Department of Psychology, University of Amsterdam, Amsterdam, The Netherlands

## Abstract

Humans typically make several saccades per second. This provides a challenge for the visual system as locations are largely coded in retinotopic (eye-centered) coordinates. Spatial remapping, the updating of retinotopic location coordinates of items in visuospatial memory, is typically assumed to be limited to robust, capacity-limited and attention-demanding working memory (WM). Are pre-attentive, maskable, sensory memory representations (e.g. fragile memory, FM) also remapped? We directly compared trans-saccadic WM (tWM) and trans-saccadic FM (tFM) in a retro-cue change-detection paradigm. Participants memorized oriented rectangles, made a saccade and reported whether they saw a change in a subsequent display. On some trials a retro-cue indicated the to-be-tested item prior to probe onset. This allowed sensory memory items to be included in the memory capacity estimate. The observed retro-cue benefit demonstrates a tFM capacity considerably above tWM. This provides evidence that some, if not all sensory memory was remapped to spatiotopic (world-centered, task-relevant) coordinates. In a second experiment, we show backward masks to be effective in retinotopic as well as spatiotopic coordinates, demonstrating that FM was indeed remapped to world-centered coordinates. Together this provides conclusive evidence that trans-saccadic spatial remapping is not limited to higher-level WM processes but also occurs for sensory memory representations.

## Introduction

To only process visual information while it is available to the eyes would be a fatal disadvantage for dynamic agents in a dynamic world. A memory buffer allows us to access visual information after termination of its retinal input (visuospatial short-term memory; VSTM). Aristotle already mentioned the phenomenon of visible persistence in 3rd century B.C.^1^, and the scientific study of VSTM goes back to at least Fechner^2^ and Helmholtz^3^. It continues to captivate researchers to this day^4^. The discrepancy between the rich subjective experience of the world and the scientific evidence for the limits of perception is especially puzzling^5^.

The most commonly studied form of VSTM, stable visuospatial working memory (WM) is robust to visual masking^6^, can last tens of seconds but has a very limited capacity^7^ and requires dedicated attentional resources^8,9^. The contents of VSTM are typically probed in change-detection experiments, which require observers to compare two arrays of visual stimuli that are presented in succession. However, memory traces that exist beyond the capacity of WM, so called sensory memory, are easily disrupted by new visual input. VSTM is thus composed of stable WM and unstable sensory memory. In traditional change-detection experiments, sensory memory traces are abolished by the presentation of the second array (i.e., the memory probe), such that only robust WM can be measured. The partial-report paradigm^10^ and the use of retro-cues^11,12^ have enabled researchers to investigate forms of sensory memory, such as iconic memory^13^ (IM): an unstable, pre-attentive, high-capacity memory trace that lasts a few hundred milliseconds. Prior to probe onset, a retro-cue indicates which items will be tested and directs internal attention (cognitive resources) to this item’s memory representation. In this way an unstable memory trace can be stabilized, protected from being masked by the probe, and subsequently reported (for a review of the retro-cue paradigm see Souza and Oberauer^14^). This procedure dramatically increases capacity estimates because now both stable and unstable memory can be measured. Recently, a second form of sensory memory has been proposed to exist as an intermediate stage between IM and WM: fragile memory (FM)^15^. FM lasts several seconds and has a capacity that lies between IM and WM^15^. FM appears to be largely unimpaired by a withdrawal of attentional resources^16,17^, which has led to the proposal that FM represents pre-attentive memory. At the very least, and in contrast to items in WM, FM items that can only be reported in a retro-cue paradigm have received sufficiently fewer attentional resources to be masked by the visual interference of a probe array. While IM is masked by any visual stimulation such as a light flash or noise pattern, FM is only masked by visual input that resembles the memorized object ^6^. This suggests that FM, unlike IM, is object-based: visual features (e.g. orientation) are bound to locations. FM has been demonstrated for both simple stimuli as well as more complex objects^15^. Whether FM really is a separate form of memory remains under debate^18^. We refer to FM as simply the increased capacity observed in the retro-cue paradigm and assume these additional items to have received little to no attentional resources. It is so far unknown whether FM survives eye movements, which forms the focus of the present study.

Humans typically make several fast eye movements (saccades) per second. This provides a challenge for the visual system as locations are largely coded in retinotopic (eye-centered) coordinates. To retain the actual location of an item, its memory representation needs to be updated with every saccade. Spatial remapping is the updating of retinotopic coordinates to task-relevant, spatiotopic (world-centered) coordinates^19^. It is so far unknown whether high-capacity sensory memory is remapped across saccades. The visual system could preserve resources by remapping only few items. Indeed, influential accounts of spatial remapping advocate that an item must have first received dedicated attentional resources before it can be remapped^20–22^. This view proposes that only items in stable WM are maintained across eye movements. Reports from trans-saccadic VSTM experiments have suggested that with every saccade or other visual interruption we lose all information about the world except for what was actively attended. Investigating trans-saccadic IM, Irwin^23^ discovered that the number of letters they remembered scaled with set size when participants made no eye movements. After saccades, capacity remained at a ceiling of three to four items regardless of set size. Irwin concluded that while stable WM is maintained across eye movements, IM is not. Prime *et al*.^24^ also reported a trans-saccadic capacity limit of three to four items for luminance and orientation, and noted that attended items were more likely to be remembered. This evidence seems to suggest that much of the initial representation of the visual world is lost after saccades.

The observed capacity limit of trans-saccadic memory, however, stands in stark contrast to our subjective experience of the world, which appears detailed, stable and continuous across saccades. There is evidence for the retention of highly detailed information across saccades, such as precise location information. Peri-saccadic displacement of a saccade target typically goes unnoticed, even if the displacement is large^25^. However, if the target is removed for a short time after the saccade has landed, even very small displacements can be noticed. Spatiotopically presented masks have also been found to reduce the capacity of sensory memory, suggesting that in addition to un-maskable WM, sensory memory representations also exist in spatiotopic coordinates^23,26^. Germeys *et al*.^27^ previously reported an increase in trans-saccadic memory capacity when using a retro-cue followed by a blank as compared to not using a blank. However, their paradigm did not allow to distinguish in which state of memory (stable or fragile) the items existed at the time of remapping. To investigate remapping of purely sensory memory the saccade target in the present study was placed outside of the memory array.

**Figure 1:**
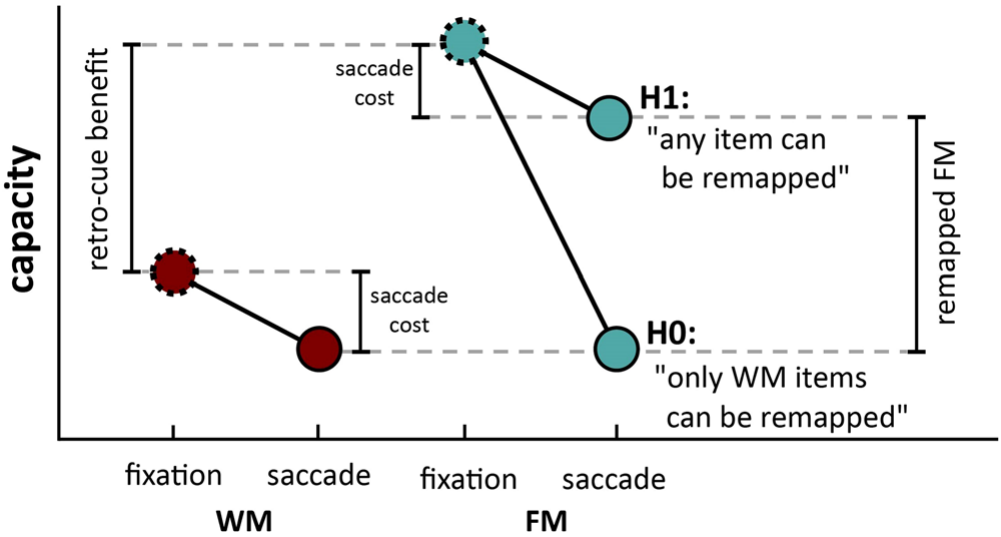
Hypotheses examined in Experiment 1. The retro-cue benefit demonstrates a higher memory capacity when using a retro-cue (i.e. FM). If this difference is also observed in the saccade condition, then this demonstrates trans-saccadic FM. Dashed circles indicate within-fixation capacity, circles with a solid outline indicate across-saccade capacity.

The present study investigated whether sensory memory is remapped to world-centered coordinates. For this purpose, we employed a retro-cue change-detection paradigm and required observers to make a saccade during the retention interval. We ask the question whether only items in robust WM are maintained across saccades or whether items in sensory memory are also maintained. If FM is remapped, then trans-saccadic memory capacity in the retro-cue condition (tFM) should be higher than trans-saccadic memory capacity in the post-cue condition (tWM). The capacity difference indicates the number of remapped sensory memory items (Fig. 1). A small performance drop is expected in the saccade conditions relative to fixation conditions as the saccade target typically takes up about one item in memory^28^. The results clearly support the hypothesis that sensory memory items have been remapped. In a second experiment, we presented backward masks to selectively disrupt memory representations in retinotopic or spatiotopic coordinates to investigate where and when FM representations exist across saccades. The purpose of the masks in this experiment was not to interfere with stimulus encoding, but to interfere with the memory signal of the stimulus, which is required for subsequent report. These masks can be thought of as interfering items, which replace fragile memory traces encoded in the locations in which they are presented. We observed both retinotopic and spatiotopic masks to be effective. This confirms that FM items do not only exist in retinotopic coordinates but are also remapped the spatiotopic coordinates.

## Methods

### Participants

All participants reported normal or corrected to normal visual acuity and gave informed consent. 8 Utrecht University students (aged 20–26, 6 female) participated in Experiment 1 and 21 students (aged 19–41, 15 female) participated in Experiment 2 for monetary reward. The study was approved by the Ethics Committee of the Faculty of Social and Behavioral Sciences of Utrecht University and has been carried out in accordance with the Declaration of Helsinki.

### Materials

Stimuli were displayed in a dark room on an ASUS PG278q 27′′ LCD monitor with a display area of 60 × 34 cm (49.6 × 29.3 dva; degrees visual angle) and a resolution of 2560 × 1440 px at a refresh rate of 100 Hz and response time of 1ms. Monocular eye movements were recorded by an Eyelink1000 eye tracker (SR Research Ltd, Canada) at a temporal resolution of 1000 Hz and a maximal spatial resolution of 0.01 dva. Participants were seated on an adjustable chair and placed their head on a chinrest 65 cm in front of the screen. The experiment was designed in Matlab 2015a and Psychtoolbox 3^29,30^.

### Stimuli and procedure

#### Experiment 1

Following verbal and on-screen instructions, participants completed the task with short breaks, approximately every 10–15 minutes. The eye tracker was re-calibrated at the beginning of the session and after each break. To ensure that the stimuli elicited no retinal afterimages we calibrated the gray value of the screen background to be perceptually isoluminant to the red (2.71 cd/m^2^, x = 0.646, y = 0.338) stimuli for every participant. This was done by means of heterochromatic flicker photometry^31^.

**Figure 2:**
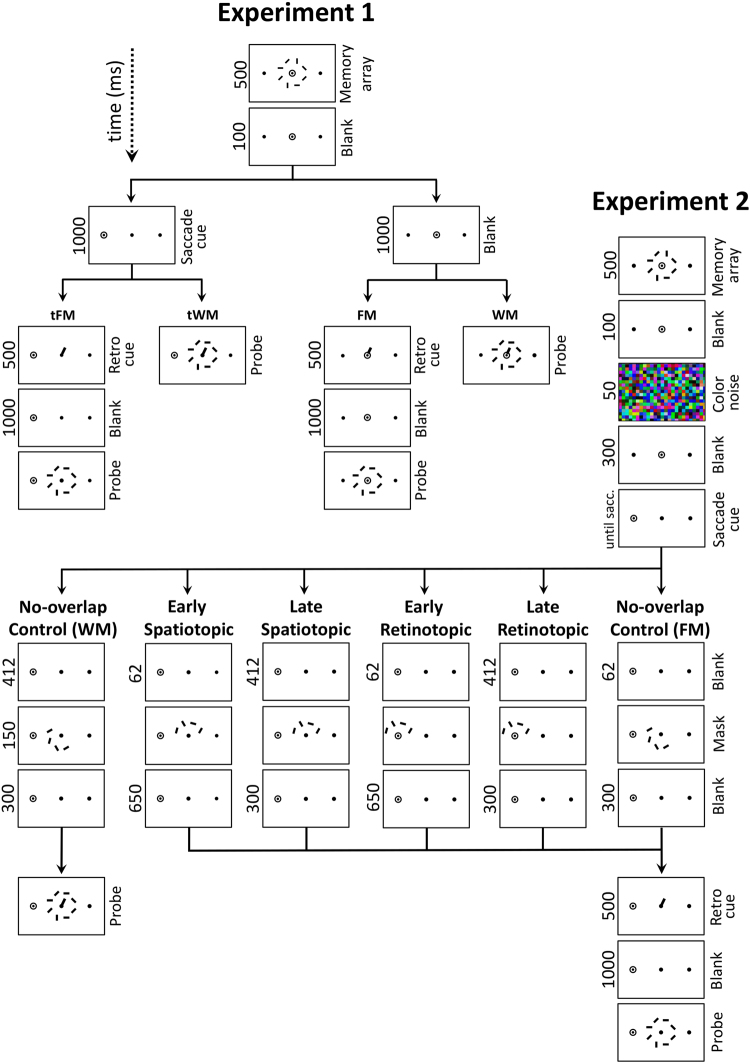
Schematic representation of trials in each experimental condition in Experiment 1 and 2. Red dots represent the currently active fixation. Sizes not to scale and colours modified for clarity. Every trial began with an array of oriented bars, the stimuli to be memorized. This was followed by a blank or a saccade cue. A retro-cue followed by a blank and a probe array was presented in the FM conditions, a probe display was presented in the WM conditions. In Experiment 2 a colour noise mask was presented after the memory array and different masks were presented after the saccade.

Each trial began with three dots (0.22 dva) that always remained visible. The central fixation dot was flanked by two dark gray dots at a horizontal distance of 6.2 dva and remained blue until participants pressed the space bar to begin a trial, upon which the central dot turned red. The trial layout is illustrated in Fig. 2. A memory array consisting of eight red rectangles in one of four orientations (0.25 × 0.9 dva, 2.5 dva from central fixation) was presented for 500 ms (Fig. 2). On half the trials, after a blank interval of 100 ms (only the three fixation dots were visible), the red fixation dot jumped to the left or the right with the other two dots displayed in dark gray. On the other half of the trials the central dot remained red. Participants were required to keep their gaze at the red dot and to move their eyes immediately when it jumped to a new location. When their gaze deviated 2.5 dva from the red dot the trial was aborted and repeated at the end of the block. Participants subsequently pressed the space bar to go to the next trial. On half the trials, after 1000 ms to allow for both saccade execution and remapping, a valid retro-cue appeared as a red line (0.05 dva in width) from central fixation extending 1.44 dva in the direction of one of the memory items. After another blank of 1000 ms the probe array appeared again with either no change or with one of its items rotated 90°. This was always the cued item. Participants were instructed to indicate whether or not they noticed a change in the cued item by pressing the up/down arrow keys. After each trial participants received auditory feedback. The time delay between memory array offset and retro-cue (the delay interval at which FM capacity is probed) was 1100 ms. On the other half of trials, the cue appeared together with the probe array (post-cue). This produced four conditions: post-cue with and without intervening saccade (i.e., probing WM), retro-cue with and without intervening saccade (i.e., probing FM). Crucially, the cue in the retro-cue condition appeared at the same time as the probe array in the post-cue condition, ensuring that WM and FM capacities could be adequately compared. Change/no-change as well as left/right saccade cue were balanced between conditions.

#### Experiment 2

See Fig. 2 for a trial flow diagram. The second experiment was very similar to the first with three distinctions. First, masks were displayed after the saccade and before the retro-cue in order to selectively disrupt FM representations. These masks had the form of four randomly oriented bars, presented for 50 ms, followed by a blank for 50 ms and then followed by a second set of four randomly oriented bars for 50 ms. The masks overlapped the location of the future target in either a retinotopic location (around the current fixation dot) or a spatiotopic location (around the central dot). Presentation was either 62 ± 5 ms (early) or 412 ± 5 ms (late) after the saccade. The post-mask blank was presented for 650 ms (in the early condition) or 300 ms (in the late condition) after the mask to keep the delay interval identical between early and late masks. In two control conditions (tFM and tWM) masks were presented 400 ms after the saccade, in a spatiotopic location, containing no overlap with the future target. This ensured that a masking effect couldn’t be explained by an increased working memory load. Second, five frames of colour noise were presented after the memory array, followed by a 300 ms blank to further eliminate the possibility that the effects observed in Experiment 1 could be explained by retinal afterimages. Third, the saccade cue remained on screen until gaze was detected 2.5 dva outside previous fixation. If no saccade was detected in this way after 500 ms the trial was aborted and repeated at the end of the block. This resulted in an average cue delay of 1312 ms + 222 ms (median saccade latency), almost half a second longer than in Experiment 1.

### Data Analysis

Based on Cowan’s *k* ^7^ we estimated memory capacity as:

1 $$k=[2\,\times \,accuracy\,-\,1]\,\times \,memory\,set\,size$$

It should be noted that the present study is agnostic with regard to working memory resources being allocated according to a discrete slot^32^ or continuous resource^33^ model, a subject of ongoing debate. Cowan’s *k* here can be seen as a continuous measure of capacity that is independent of set size. The term items in the present article therefore simply refers to a point on the Cowan’s *k* scale. The essential metric is the difference in capacity between conditions within participants, rather than an absolute estimate of capacity limits in terms of number of discrete items.

#### Experiment 1

Sessions lasted 1.5 h in which participants performed 470 experimental trials on average, about 118 per condition. Participants practiced the memory task without the saccade condition for 1.5 h on the previous day.

Previous studies investigating FM have found that about one quarter of participants require excessive training or are unable to learn the task at all. It should be noted that this does not necessarily reflect individual differences in FM but more likely a failure to use the retro-cue, which requires participants to translate the exogenous retro-cue into an endogenous shift of attention. Since the focus of the present study was not within-fixation FM, two participants who did not supersede the pre-determined accuracy threshold of 85% in the within-fixation FM condition at the end of the training session were excluded from further participating in the experiment. This ensured reliable data for addressing the research question at hand: trans-saccadic FM.

#### Experiment 2

Sessions lasted 2 h, during which participants performed 530 experimental trials on average, about 88 per condition. Participants practiced the task without masks for 2 h on the previous day. In line with Experiment 1, 7 participants were excluded during the training session if they did not reach 85% accuracy in the within-fixation FM condition after three blocks.

### Statistical analysis

Bayesian analysis allows the use of a stopping rule^34^. Data was collected until a preliminary analysis via a directional Bayesian t-test (JASP Team, 2017; JASP Version 0.8.3.1) comparing the relevant conditions (tWM, tFM) reached BF_+0_ > 6 or BF_+0_ < 1/6 (Experiment 1), or after two months of data collection (Experiment 2). A one-sided test rather than a two-sided test was used since there was a strong expectation that tFM is larger than tWM. Subsequent analyses were performed in Stan^35^ using R^36^ and the R package brms^37^. The model was a multilevel logistic regression using random intercepts to control for individual baseline differences with model equation

2 $${\rm{logit}}(respons{e}_{ij})={b}_{0}+{u}_{i}+\sum _{con=1}^{{n}_{con}}{b}_{con}{x}_{con,j}\,$$

where $respons{e}_{ij}$ is the predicted binary response (success) of participant $i$ to trial $j$, ${b}_{0}$ is an intercept, which coincides with the mean log odds for the reference category, ${u}_{i}$ is the random intercept for participant $i$, ${n}_{con}$ is the total number of non-reference conditions, ${b}_{con}$ is a difference parameter for condition *con* and ${x}_{con,j}$ is a dummy variable indicating whether trial $j$ belongs to condition *con*.

Priors were set to be weakly informative for the first experiment. From previous research it is clear that participants will almost certainly do better than chance and will almost certainly not achieve perfect scores, which is reflected in the priors. The second experiment used the same random intercept model with different conditions. For this experiment, more informative priors were set based on the knowledge obtained in the first experiment. Priors are described in detail in the supplementary material.

We employed the Bayesian hypothesis testing framework using Bayes factors^38^. For all hypothesis tests we first assessed the equality of two parameters, and subsequently tested for the direction of the effect^39,40^. The equality hypothesis tests (H_0_: conditions are equal; H_1_: conditions are different) were computed in brms by the Savage-Dickey method^41^. The directed hypothesis tests are based on the ratio between the proportion of the posterior that is in agreement with a hypothesis and its complement. In other words, the ratio between the probability density mass of a difference between two conditions above and below zero^42–44^. Posterior model probabilities were computed, for which prior odds for the hypothesis pairs under comparison were set to 1 to reflect equal weights of the hypotheses a priori. Analysis scripts and raw data are available via the Open Science Framework at https://osf.io/ye9ya/.

## Results

### Experiment 1

In the first experiment, we demonstrate a retro-cue benefit irrespective of whether observers made a saccade. This provides strong evidence that FM items were remapped across saccades.

#### Higher capacity in the retro-cue condition

Accuracy scores for tFM were higher than tWM scores by 9.5 percentage points on average (0.48 ± 0.1 (mean ± standard deviation) on the log-odds scale). This corresponds to an increase in memory capacity by *k* = 1.5 items. The Bayes Factor in favor of unequal tFM and tWM was 5530 with a posterior model probability of ≈1. The directed hypothesis test indicated that tFM capacity is almost certainly larger than tWM capacity (posterior model probability ≈1).

Accuracy scores in the FM condition (within fixation) were 10.8 percentage points higher than WM scores (0.69 ± 0.12 on the log-odds scale). This corresponds to an increase in memory capacity by *k* = 1.7 items. The Bayes Factor in favor of unequal FM and WM was 26982 with a posterior model probability of $\approx 1$. The directed hypothesis test indicated that FM capacity is almost certainly larger than WM capacity (posterior model probability ≈1). Comparing tFM with FM capacity suggests that on average 1.5 of 1.7 sensory memory items were remapped.

#### Summary

**Figure 3:**
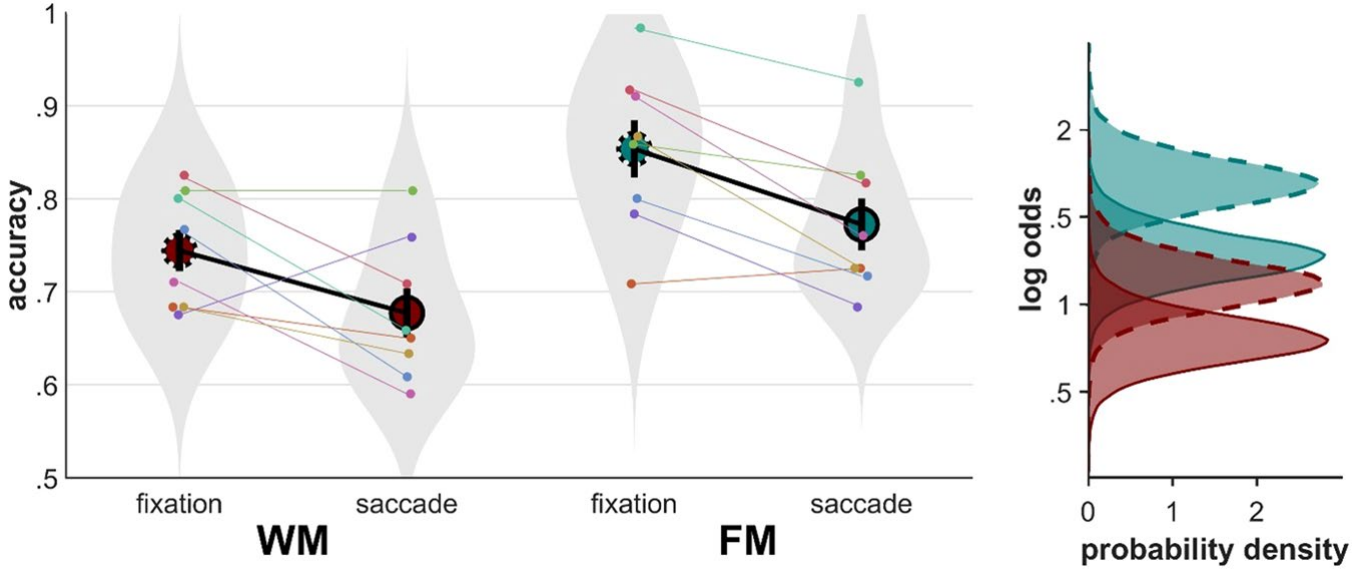
Results Experiment 1. (left) Individual proportions correct and means of proportion correct. Dashed circles indicate within-fixation capacity, circles with a solid outline indicate across-saccade capacity. Error bars represent SE. Shaded gray areas are violin plots to visualize the shape of the distributions. (right) Posterior probability densities in log odds space. As can be seen, tFM capacity (cyan) is larger than tWM capacity (red).

We provide strong evidence that in addition to stable memory items some, if not all, fragile memory items have also been remapped. Individual data is shown in Fig. 3 (left panel). The posterior probability density distributions are shown in Fig. 3 (right panel). These distributions represent our knowledge about possible values of the average proportion correct in each condition. Plots displaying the hypothesis tests graphically are provided in the supplementary material.

### Experiment 2

In the second experiment, we selectively masked locations to disrupt FM representations in order to assess where in space and time FM exists across saccades. The informative priors employed in this section are discussed in the supplementary materials. The results show that masks at the retinotopic location as well as at the spatiotopic location reduced the retro-cue benefit. This confirms that FM items have been remapped to task-relevant, world-centered coordinates.

#### Higher capacity in the retro-cue condition

First, we replicated the main finding of Experiment 1. On average, tFM accuracy scores were 10.1 percentage points higher than tWM scores (0.51 ± 0.07 on the log-odds scale). The Bayes Factor in favor of unequal tFM and tWM was 28408, with a posterior model probability of ≈1. The directed hypothesis test again indicated that tFM capacity is almost certainly larger than tWM capacity with a Bayes Factor approaching infinity and posterior model probability ≈1.

#### No difference between early and late masks

For retinotopic masks, the Bayes Factor was 4.3 in favor of no difference between early and late mask presentation, with a posterior model probability of 0.811. Retinotopic early masks were 1.5 percentage points more effective than retinotopic late masks. For spatiotopic masks, the Bayes Factor was 7.5 in favor of no difference between early and late mask presentation, with a posterior model probability of 0.883. Spatiotopic late masks were 0.3 percentage points more effective than spatiotopic early masks. Following this evidence for no difference, early and late mask data were pooled for retinotopic and spatiotopic mask conditions respectively.

#### Retinotopic and spatiotopic masks are effective

On average, accuracy scores in the retinotopic mask condition were 4.8 percentage points lower than tFM scores (0.25 ± 0.06 on the log-odds scale). The Bayes Factor in favor of unequal tFM and retinotopic mask conditions was 600, with a posterior model probability of 0.998. The directed hypothesis test indicated that retinotopic masks almost certainly were effective with a Bayes Factor approaching infinity and posterior model probability ≈1.

On average, accuracy scores in the spatiotopic mask condition were 3.6 percentage points lower than tFM scores (0.19 ± 0.06 on the log-odds scale). The Bayes Factor in favor of unequal tFM and spatiotopic mask conditions was 16.84, with a posterior model probability of 0.944. The directed hypothesis test indicated that spatiotopic masks almost certainly were effective with a Bayes Factor of 1999 and a posterior model probability ≈1.

#### Summary

**Figure 4:**
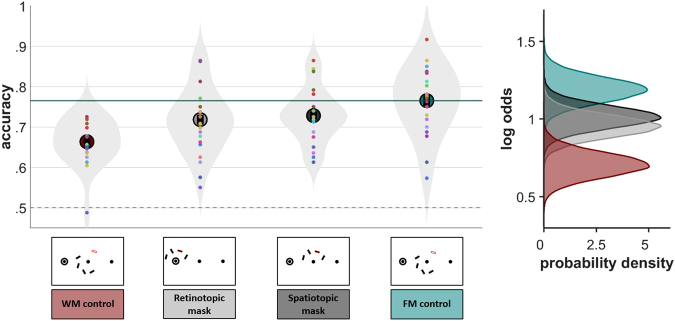
Results Experiment 2. (left) Proportions correct per participant and means of proportions correct per condition. Dashed circles indicate within-fixation capacity, circles with a solid outline indicate across-saccade capacity. Error bars represent SE. Shaded gray areas are violin plots to visualize the shape of the distributions. The mask type used is visualized below the violin plots of each condition. The transparent bar indicates the future target location. (**right**) Posterior probability densities in log odds space, which provide strong support that both spatiotopic and retinotopic masks interfered with memory representations (difference between cyan and gray distributions), as well as a replication of Experiment 1 (difference between cyan and red distributions).

We confirm that (1) tFM capacity is larger than tWM capacity and (2) FM is remapped to spatiotopic coordinates. Individual data is shown in Fig. 4 (left panel). The posterior probability densities, which represent our knowledge about possible values for the parameters and display the effects found graphically, are shown in Fig. 4 (right panel). Plots displaying the hypothesis tests graphically are provided in the supplementary material.

## Discussion

We investigated trans-saccadic visuospatial short-term memory (tVSTM) in a retro-cue change-detection paradigm and observed remapping of both robust and fragile memory traces. In addition to items that received dedicated attentional resources (robust WM), some items in unstable, pre-attentive FM were also remapped. Our results challenge the strongly held beliefs that (1) remapping always requires attentional resources, and (2) that tVSTM contains only WM items and thus is synonymous with tWM.

### The capacity of trans-saccadic VSTM

Previously, tVSTM capacity has been estimated to underlie the same limitations as WM: three to four items^23,24^. According to this account, items that did not receive sufficient attentional resources prior to the saccade are lost after the saccade. We argue that studies investigating tVSTM have suffered from similar limitations as early studies of VSTM: there is more information in memory than can be reported in common paradigms^5^. Partial-report enabled the study of other forms of VSTM, such as sensory memory. Due to the fast decay of iconic memory it was only after the discovery of FM^15^, which has a lifetime of several seconds, that a paradigm could be developed to adequately estimate trans-saccadic sensory memory. Recently a study by Germeys *et al*.^27^ reported a post-saccadic retro-cue benefit. However, in their paradigm the state of memory items at the time of remapping is unclear because the saccade target was always one of the memory items. To investigate remapping of purely sensory memory the saccade target in the present study was placed outside of the memory array.

We observed a trans-saccadic sensory memory capacity well above that of tWM. This is a clear indication that some, if not all, FM items have been remapped. Due to individual differences in WM capacity it is crucial to compare capacities within-subject and within-task, that is, WM after a saccade with FM after a saccade, rather than to establish global capacity estimates such as the “magic number four”. With an appropriate baseline condition (tWM) any capacity increase observed for tFM must reflect items that were stored as FM but remained accessible in task-relevant coordinates after the saccade (see also Fig. 1). If only stable items survived saccades, then no retro-cue benefit should have been observed because there would not have been any fragile memory traces left for the retro-cue to rescue.

In our view, previous studies^23,24^ have failed to observe remapping of sensory memory because the employed paradigms were not sensitive enough to pick up these fragile memory traces across saccades. Irwin^23^ used a post-cue delayed-recall procedure and showed stimuli only once, requiring participants to begin recalling the test item from the set of memorized stimuli immediately after the post-cue. The intrusion-errors Irwin reported in Table 4 are much more pronounced for the saccade condition, which indicates difficulties to localize the test item in memory and explains the reduced performance after saccades. While Prime *et al*.^24^ used a change-detection task, they did not include a retro-cue. Sensory memory can’t be directly reported but must first be transferred to stable WM. The retro-cue change-detection task employed in the present study enabled participants to first stabilize a remapped fragile memory trace and to then compare it with the visible test item at the same spatiotopic location.

### Spatiotopic and retinotopic masking

It could have been possible that participants in Experiment 1 were able to use the retro-cue without remapping the memory items. To ensure that FM was indeed remapped to spatiotopic coordinates we presented masks after the saccade and before the retro-cue in Experiment 2 and found both retinotopic and spatiotopic masks to be effective, in line with previous studies^23,26^. Pinto *et al*.^6^ previously demonstrated that object-specific masks interfere with FM but not with WM, providing a tool for assessing the spatial properties of FM, namely, whether it is coded in a retinotopic or spatiotopic reference frame. Crucially, in the present study the masking effect was calculated relative to a condition with the same masking stimuli presented at locations that did not overlap with the future target. Since the masking stimuli were presented in all conditions and the only difference being their precise spatial location the masking effect cannot be explained by additional working memory load, which was a possibility in earlier studies^23,26^ or an interference with the comparison process in the change-detection task. The effectiveness of spatiotopic masks, together with the observed retro-cue benefit after the saccade, provides conclusive evidence that FM does not only survive eye movements but is indeed remapped to world-centered, task-relevant coordinates. Surprisingly, there was no difference between presenting masks early or late after the saccade. We expected primarily early retinotopic masks and late spatiotopic masks to be effective as presumably memory representations first exist in retinotopic coordinates and are then remapped to spatiotopic coordinates. However, similar patterns of results have previously been reported. Golomb, Chun and Mazer^45^ observed a lingering retinotopic trace of attentional pointers after a saccade, although for a much shorter duration than what was observed in the present study. In contrast to Golomb *et al*., who reported lingering retinotopic attentional activation, we observed an apparent lingering retinotopic memory representation. Conversely, while build-up of spatiotopic representations is often thought to be a slow process^46^, immediate build-up has also recently been observed^47^.

### Pre-attentive spatial remapping and the functional role of sensory memory

Traditionally, trans-saccadic memory was believed to be comprised of WM items only since the spatial remapping process is thought to strongly depend on attention. Attention here refers to the allocation of a limited cognitive resource to a small set of objects. Items in WM have been prioritized by attentional mechanisms, while items in FM may have received no attention at all or sufficiently fewer resources to remain in a sensory memory state, which is easily masked by visual interference^16,17^.

Especially the failure to detect a role for sensory memory in trans-saccadic perception has led this memory form to be dismissed as an artefact of visual processing^48^. By demonstrating remapping of sensory memory items that have not received dedicated cognitive resources, we confirm that sensory memory is not merely an artefact, but an object-based form of memory that is maintained and updated across eye movements. This suggests potential functional roles for sensory memory. One possibility is that trans-saccadic perception relies on a highly detailed pre-categorical representation of the world. It is conceivable that remapping an entire scene across eye movements provides a reference frame for trans-saccadic integration and a computationally efficient way to localize the most relevant objects in memory (WM) within this frame. In that case, sensory memory would support trans-saccadic visual stability, one of the great puzzles of visual neuroscience^49^.

An important question concerns whether FM items (defined as items that can only be reported in a retro-cue paradigm) depend on qualitatively different resources (i.e., a different memory store) or on quantitatively different resources (i.e., receiving less attention) than WM items. We interpret our results within a framework that assumes the former possibility (FM and WM rely on a separate cognitive resource). Two aspects would be necessary to substantiate this framework: **(1)** a differential effect of withdrawal of attention on WM and FM. **(2)** Separate neural substrates of the two memory forms.

**(1)** Vandenbroucke, Sligte & Lamme^16^ found that reducing available attentional resources affected WM and FM very differently. They conducted three experiments to withdraw attention during a change detection task. Temporal uncertainty, a parallel n-back task and an attentional blink paradigm all demonstrated differential effects of attention withdrawal on WM and FM. Whereas WM capacity was reduced by withdrawing attention, FM capacity was reduced only minimally. The authors (or we) do not argue that FM operates independently of neural resources, as that would preclude any brain activity, but that FM operates independently of the resource that WM depends on (i.e. “attention”). Crucially, qualitative differences in capacity reduction between WM and FM were observed: a 2-back task impacted WM stronger than a 1-back task. This was not the case for FM. In other words, the accuracy reduction did not scale with the level of attentional diversion. The attentional blink impacted WM depending on lag position. FM was impacted identically at any lag position. In other words, the impact on FM was likely driven by the dual-task rather than the attentional blink as such. The observed numerical and qualitative differences make a strong case for WM and FM relying on separate cognitive resources and FM being a pre-attentive form of VSTM. Vandenbroucke *et al*.^16^ argued that FM represents the initial storage capacity of VSTM while WM depends on attentional boosting of some of these items for cognitive manipulation and report. This conclusion is in line with the initial study by Landman *et al*.^11^, who demonstrated that even after an item had initially been attentionally selected by a retro-cue, a benefit from a second retro-cue, selecting a different item from the memory set, could still be retrieved. Furthermore, Pinto *et al*.^17^ observed a greater performance reduction in WM than FM when spatial attention was withdrawn from the future target during encoding. They presented 80% valid pre-cues before the memory array and examined the invalidly cued trials to measure the effect of attentional resource withdrawal. They concluded from the pattern of results (constant performance reduction in both paradigms) that the WM component was affected in both cases, indicating that WM and FM are separate rather than redundant memory stores.

**(2)** Sligte, Wokke, Tesselaar, Scholte and Lamme^50^ discovered that TMS pulses to the right DLPFC strongly affected measures of WM but not FM. Sligte *et al*. were asking the question whether FM is a weak form of WM (items that received less attention); or whether FM is independent of the attentional boosting that WM relies on. Their results support the hypothesis that FM does not depend on the wide-spread network activity associated with the attentional boosting observed in WM, but relies at least partially on a different neurological architecture.

Taken together these results strongly suggests that FM does not depend on attention in the way WM does and that FM items received little to no attention during encoding. Irrespective of whether or not the FM interpretation of the retro-cue benefit effect holds, the present results at the very least demonstrate that items that exist in a lower priority state (fewer cognitive resources, less attentional prioritization, high maskability) are remapped in addition to those items that were prioritized and received most resources.

## Conclusion

In summary, the present study provides compelling evidence for spatial remapping of items in pre-attentive, high-capacity, fragile sensory memory across eye movements. This contradicts previous studies that confine remapping to robust, capacity-limited WM and has implications for the role of attention in spatial remapping.

## Electronic supplementary material

Supplementary material
