# Supplementary material for: Remapping high-capacity, pre-attentive, fragile sensory memory
Source: Sci Rep. 2017 Nov 21;7:15940. doi: 10.1038/s41598-017-16156-0 (PMC5698417; doi:10.1038/s41598-017-16156-0)
Supplement: Supplementary file 1 — Supplementary material [file 41598_2017_16156_MOESM1_ESM.pdf]

Remapping high-capacity, pre-attentive, fragile sensory memory.

Paul Zerr, Surya Gayet, Kees Mulder, Yaïr Pinto, Ilja Sligte, Stefan Van der Stigchel

Supplementary material

I. Prior specification

Priors were set to be weakly informative for Experiment 1. From previous research it is clear that participants will almost certainly do better than chance, as well as almost certainly not achieve perfect scores. This was reflected in the priors by placing an N(1, 4) prior on the intercept on the log odds scale, and putting an N(0, 1) prior on each of the group differences. The prior for the standard deviation of the distribution of intercepts was set automatically uninformative by the default procedure in *brms*.

Bayesian analysis allows us to include the obtained results from the first experiment as prior knowledge, by setting the previously obtained posterior as the prior for Experiment 2. This is widely viewed as one of the major advantages of the Bayesian framework. Setting informative priors is possible here for tWM and tFM, which differ only slightly between experiments. However, because of these slight differences, we prefer to remain conservative about our prior knowledge. Therefore, we multiplied the posterior variances by 2, which allowed potential differences between the two experiments to be found more easily. Specifically, our prior for the reference group, tFM, was N(1.216, 0.322), and for the difference parameter between tFM and tWM N(-0.481, 0.204).

For the new masking conditions, we set weakly informative subjective priors. From the literature it is clear that accuracy in a masking condition will be lower than in tFM and higher or close to tWM. This is reflected in an N(-0.24, 0.4) prior for the difference between tFM and each masked condition.

The plot below displays the prior for the mean of the retinotopic and spatiotopic masks, alongside the lower bound in tWM and upper bound in tFM taken from Experiment 1 (vertical bars). This prior reflects our belief that the probability of success for the masked conditions falls in between tWM and tFM and that we are unsure as to where in between these results will fall.

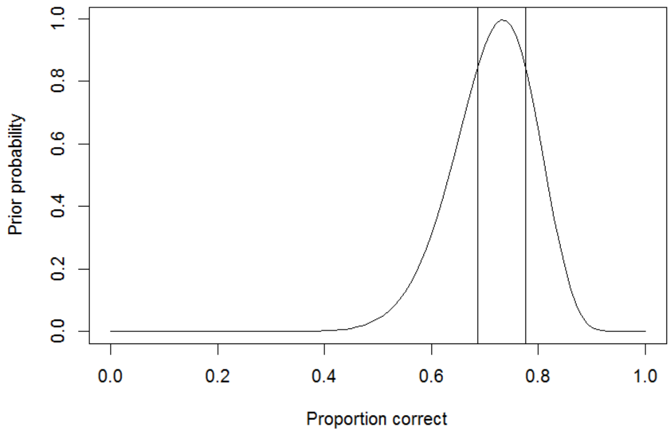

Supplementary Figure 1 | Informative prior used in Experiment 2. Vertical lines indicate lower bound for tWM and upper bound in tFM taken from Experiment 1.

II. Hypothesis tests

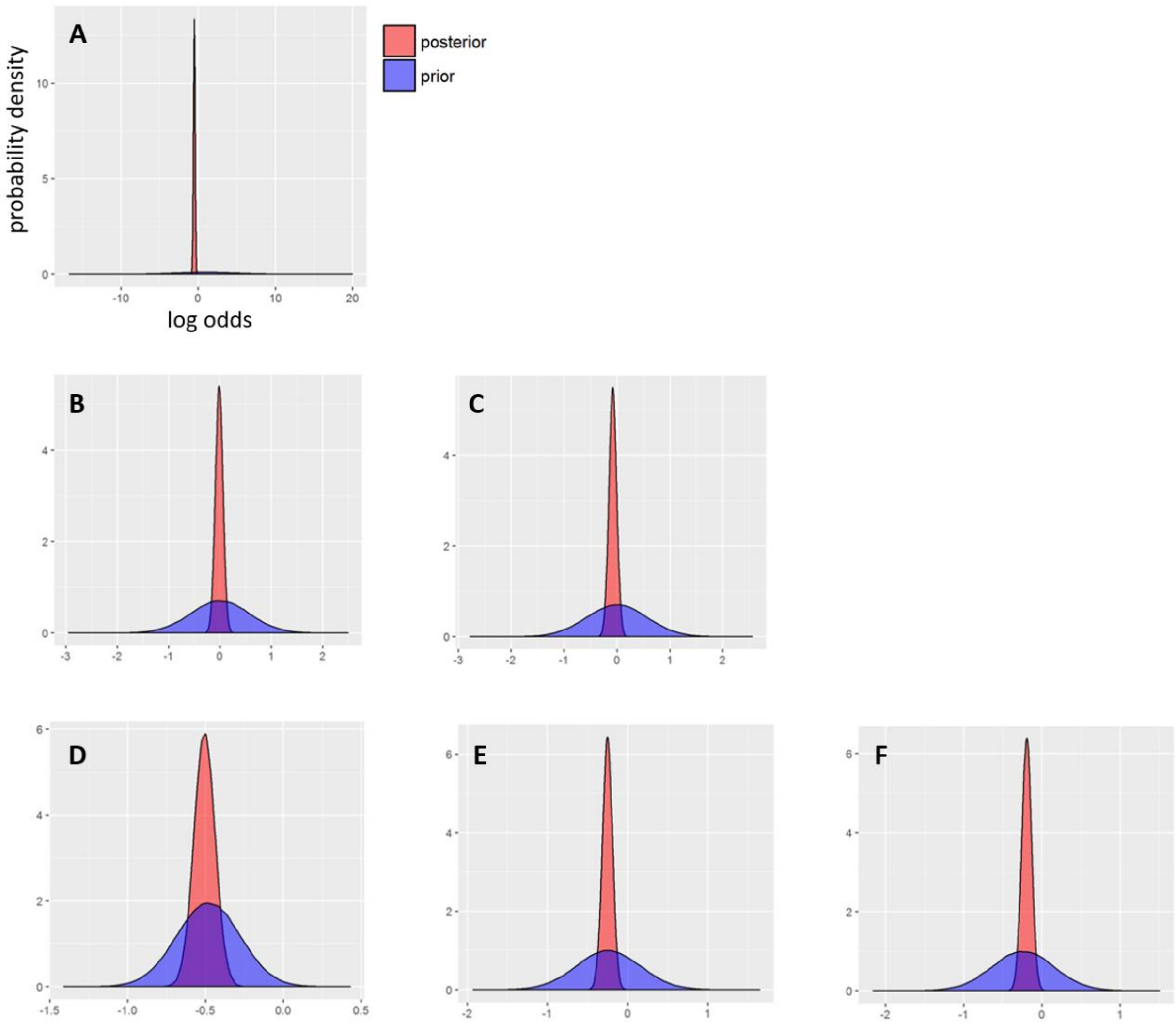

Supplementary Figure 2 | Prior and posterior distributions of the testing quantity for each Bayesian hypothesis test in log odds space. (A) Experiment 1: Accuracy difference between tFM and tWM. **(B)** Experiment 2: difference between early and late spatiotopic mask effect. **(C)** Experiment 2: difference between early and late retinotopic mask effect. **(D)** Experiment 2: difference between tFM and tWM. **(E)** Experiment 2: difference between retinotopic mask effect and FM control condition. **(F)** Experiment 2: difference between spatiotopic mask effect and FM control condition.

III. Sequential analysis plot

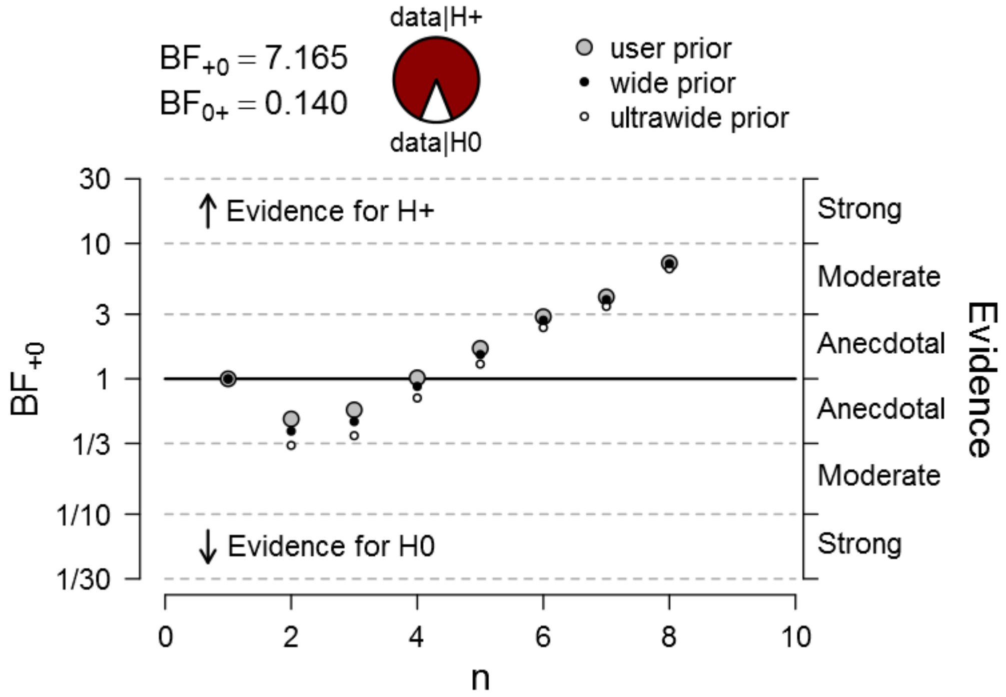

Supplementary Figure 3 | Sequential analysis plot Experiment 1. Development of the Bayes factor in Experiment 1 with each additional subject in a directional Bayesian t-test, which triggered the stopping rule at $BF_{+0} > 6$.
